# Fumarate hydratase-deficient renal cell carcinoma complicated with liver metastasis: case report

**DOI:** 10.3389/fsurg.2024.1430344

**Published:** 2024-11-01

**Authors:** Hanmin Chen, Qingming Zeng, Folin Liu, Xiaofeng Zou

**Affiliations:** Department of Urology, The First Affiliated Hospital of Gannan Medical University, Ganzhou, China

**Keywords:** FH-RCC, liver metastasis, surgery, targeted therapy, good prognosis

## Abstract

**Background:**

Fumarate hydratase-deficient renal cell carcinoma (FH-RCC) is a rare subtype of kidney tumor. Most of them are solitary lesions, making preoperative diagnosis difficult, aggressive, and with poor prognosis. They may metastasize even at an early stage, however, there is currently no optimal diagnostic and therapeutic approach for metastatic FH-RCC.

**Methods:**

We report the case of a 34-year-old male patient with renal tumor and liver metastasis, who underwent open tumor resection of the right kidney combined with resection of liver metastases. Postoperative pathology was confirmed, followed by targeted therapy.

**Results:**

Postoperative pathological results confirmed FH-RCC, targeted therapy was performed after surgery. No tumor recurrence was observed during the follow-up of almost 16 months.

**Conclusion:**

FH-RCC patients with liver metastasis can achieve a good prognosis through early resection of primary tumor and metastatic lesions combined with targeted therapy.

## 1 Introduction

FH-RCC is a rare autosomal dominant genetic disease that occurs primarily in young and middle-aged people. The incidence of FH-RCC remains unclear and is mainly reported in individual cases. About 300 FH-RCC families have been reported in the literature (1). The improvement of diagnostic techniques has led to increased incidence rates. FH-RCC is more prone to recurrence and metastasis than other renal malignancies. The most common sites of metastasis are lymph nodes in the chest and abdomen, bone and liver and the prognosis is poor. We report a case of FH-RCC with liver metastasis, succeeding a good response with combined treatment, allowing an in-depth understanding of this rare tumor.

## 2 Case presentation

**Figure 1:**
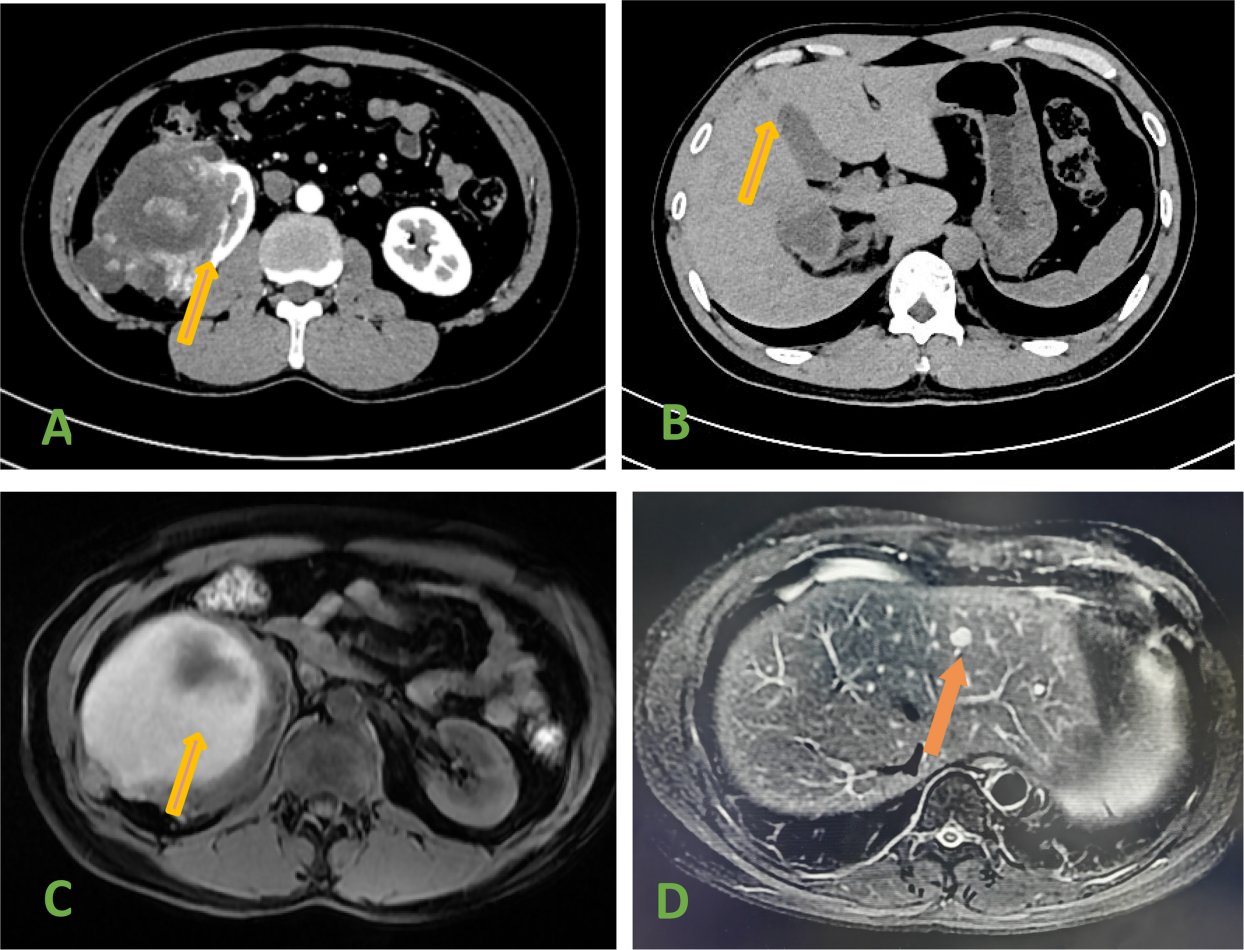
**(A)** Ct scan showed a tumor in the right kidney. **(B)** CT scan showed hepatic metastases. **(C)** MRI showed a tumor in the right kidney. **(D)** MRI Showed hepatic metastases (arrow).

The reporting of this study conforms to CARE guidelines (2) and informed consent was obtained from the patient. A 34-year-old man was admitted to the hospital due to right low back pain for 5 days. The patient had no hematuria. There was no special medical history or clear family history of the tumor. Physical examination revealed a right upper abdominal bulge, a mass under the right costal margin, and percussion pain in the right renal region. Urinary system computed tomography (CT) showed: (1) Renal tumor was found in the right kidney, with a size of 11.9 × 16.5 cm. (2) Hypodense S3 and S4 liver lesions (Figures 1A,B). Magnetic resonance imaging: mixed signal shadows of T1WI and T2W1 were observed in the right renal parenchyma with a size of 10.4 × 16.5 cm, and nodular T2W1 signal shadows with a size of about 1.0 cm were observed in the left hepatic lobe (Figures 1C,D). Bilateral renal Emission Computed Tomography (ECT): left kidney GFR 60.9 mL/min, right kidney GFR 26.1 mL/min. Whole body bone imaging and chest CT showed no obvious abnormality.

Open tumor resection of the right kidney and hepatic metastasis was performed under general anesthesia. A subcostal incision was made, and the tumor was located in the upper pole of the right kidney, locally adherent to the surrounding tissue. The surface vessels were dilated. The right renal artery, vein, and ureter were ligated. The right kidney was separated and completely resected. Hepatobiliary surgeons assisted in the complete resection of liver metastases located in liver S3 and S4 segments. The blood loss was approximately 200 mL. After intensive postoperative care, the catheter was removed in 2 days and the abdominal drainage tube was removed in 3 days. The patient was discharged in 5 days.

**Figure 2:**
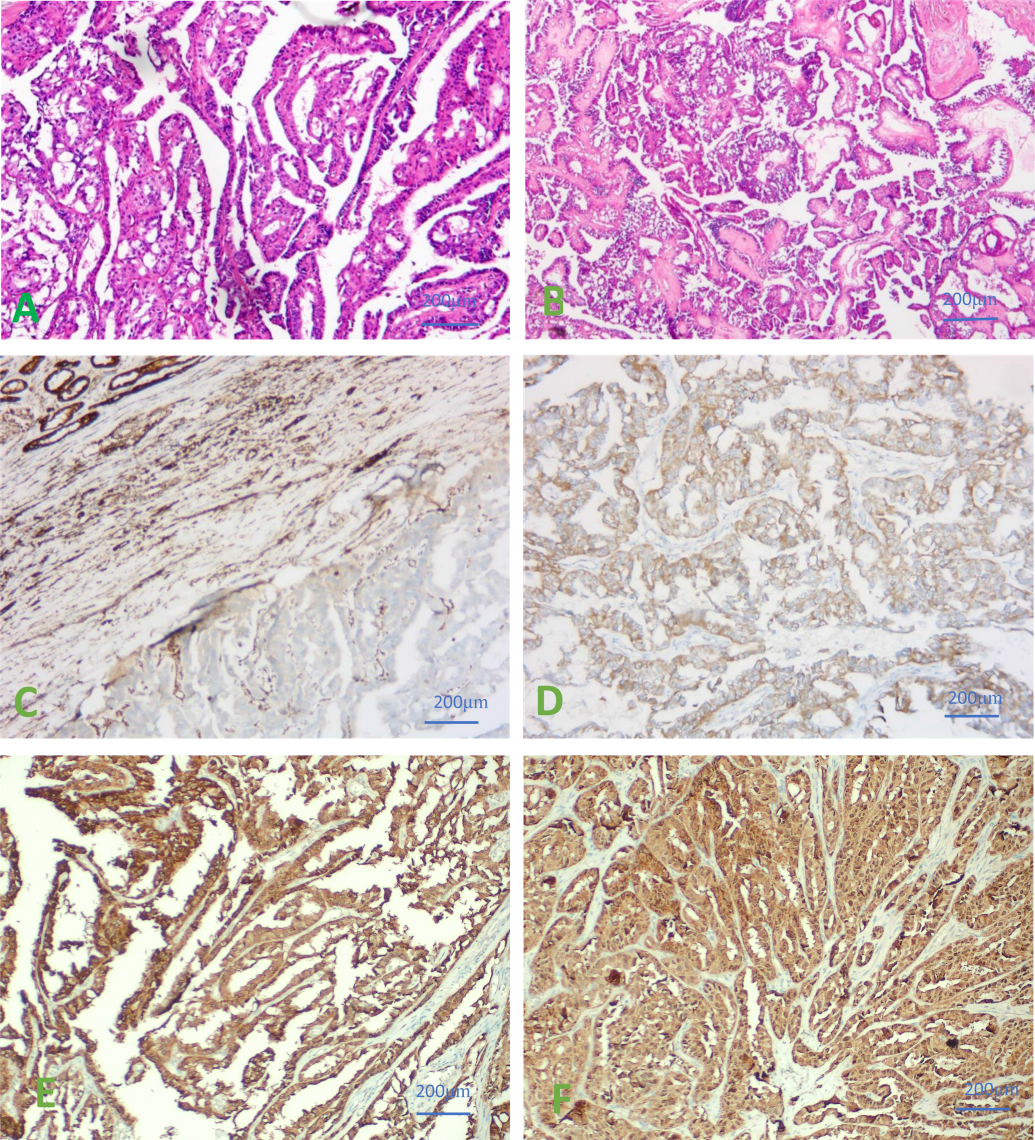
**(A)** Hematoxylin-Eosin (HE) staining of right renal cell carcinoma was seen microscopically. **(B)** HE staining of liver metastases were observed under microscope. **(C)** IHC results of negative FH staining in renal tumor cells. **(D)** IHC results of negative FH staining in liver metastases. **(E)** IHC staining of 2SC showed a strong positivity in the cytoplasm of neoplastic cells in renal tumor cells. **(F)** IHC staining of 2SC showed a strong positivity in the cytoplasm of neoplastic cells in Liver metastases. ×400.

Postoperative pathological results were the following: the surgically removed specimen was 16.0 cm × 11.0 cm in size, presented a cystic and solid pattern. The section showed a grayish-white color and contained a grayish-yellow fluid. Necrotic tissue was also present. The liver metastases were grayish white, with a size of 1.5 × 0.8 cm. Microscopically, right renal cell carcinoma, ISUP/WHO3 grade, and liver metastases were seen (Figures 2A,B). Immunohistochemical staining results showed: CK7 (−), CD10 (−), CAIX (−), Pax-8 (+), TFE3 (−), Vim (+), CD117 (−), Ki-67 (hot spot 20%), P504S (+), GATA-3 (−), HMB45 (−), Melan-A (+), SDHB (+), FH (−). Normal renal tissue, blood vessels, inflammatory cells, or other stromal cells were used as positive internal controls. If the internal control was positive, but FH staining was completely absent in the cytoplasm of the tumor cells, it was considered to be true FH-negative, and this kind of renal cell carcinoma was classified as FH-RCC. (FH-antibody, MAB-1014, Fuzhou Maixin biological Co. Ltd.). Right renal tumor showed FH-deficient renal cell carcinoma. Liver metastases also showed metastatic FH-deficient renal cell carcinoma (Figures 2C,D). 2-Succino cysteine (2SC) staining showed a strong positivity in the cytoplasm of neoplastic cells. Immunohistochemical staining of 2SC (Anti-2SC antibody, CRB2005017e/6773, Beijing Bole Biological Technology Co. Ltd.) was positive in both right renal tumor and liver metastases (Figures 2E,F). All antibody kits were purchased from Beijing Zhongshan Jinqiao Co.

Postoperative adjuvant treatment with bevacizumab and erlotinib (bevacizumab, 10 mg/kg, soluble in 0.9% NS 250 mL intravenous infusion, 1 time/14 days; erlotinib, 150 mg once a day, taken orally 2 h after meal), a total of 6 cycles. The patient had decreased appetite and leukopenia, and the symptoms were improved after symptomatic treatment. During regular reexamination of chest and abdominal CT, hematuria analysis, renal function, and follow-up for almost 16 months, no tumor recurrence was found.

## 3 Discussion

FH-RCC is a rare subtype of renal cell carcinoma characterized by mutations and functional defects in the FH gene. The clinical manifestations are highly invasive biological behaviors, with early local progression and distant metastasis prone to occur. Abdominal or thoracic lymph nodes and bone and liver are the most common sites of metastasis (3), and are connected to poor prognosis. Large eosinophilic nucleoli and peripheral halos are major histological features in the tumoral tissue (4). The complete absence of FH immunohistochemical staining in the cytoplasm of the cancer cells is helpful for the diagnosis of FH-RCC. 2SC is produced by protein threonylation in patients with FH-RCC (5). In cancerous tissues, the results of IHC staining for FH and 2SC are clinically valuable in the diagnosis of FH-RCC (6, 7), as the absence of FH and high expression of 2SC in the cytoplasm of neoplastic cells support the diagnosis of FH-RCC (8). However, the diagnosis of FH-RCC further requires molecular testing and systematic investigation of the family history. In this case, immunohistochemical results of both primary and metastatic sites supported FH-RCC. We also advised the patient to inform and ensure the screening of relatives during disease management.

The clinical management of FH-RCC is challenging. In the past, most cases were treated with primary tumor resection and lack of comprehensive treatment strategy. We believe that performing a surgical intervention on time is of great prognostic significance, and wide resection margin surgery and retroperitoneal lymph node dissection are recommended (9). Data on systemic therapy of FH-RCC are mostly limited to case reports. The analysis of a Phase II clinical trial evaluating the efficacy and safety of bevacizumab in combination with erlotinib in the treatment of FH-RCC (NCT01130519) showed an objective response rate of 65% and a median progression-free survival of 24.2 months. Based on the results of this trial, in 2018, the National Comprehensive Cancer Network (NCCN) issued guidelines recommending bevacizumab in combination with erlotinib for the treatment of patients with recurrent or advanced FH-RCC (10). Compared with papillary RCC, FH-RCC shows higher incidence rates in younger patients, a higher proportion of radical nephrectomy, and the likelihood of receiving systemic therapy (11). We recommend considering the immunotherapy integration to provide a more comprehensive treatment regimen. Recent studies (12) have confirmed genomic and transcriptomic characteristics of FH-RCC metastases, revealing its early evolutionary trajectory and bringing new hope for treatment. In this case, the patient had developed liver metastases at the time of diagnosis. Nephrectomy and liver metastasis resection were performed, and a combination of bevacizumab and erlotinib was followed. No tumor recurrence was found during a 16-month follow-up with regular review of chest and abdominal CT.

## 4 Conclusions

In general, the prognosis of FH-RCC is poor. Recently, remarkable advances have been achieved in surgery, targeted therapy, and immunotherapy. However, concerning the pathogenesis of FH-RCC, it is urgent to explore new therapeutic agents, that may extend patients’ life expectancy.

## Data Availability

The original contributions presented in the study are included in the article/Supplementary Material, further inquiries can be directed to the corresponding author.
